# Clinical and Neurophysiological Correlates of Emotion and Food Craving Regulation in Patients with Anorexia Nervosa

**DOI:** 10.3390/jcm9040960

**Published:** 2020-03-31

**Authors:** Nuria Mallorquí-Bagué, María Lozano-Madrid, Giulia Testa, Cristina Vintró-Alcaraz, Isabel Sánchez, Nadine Riesco, José César Perales, Juan Francisco Navas, Ignacio Martínez-Zalacaín, Alberto Megías, Roser Granero, Misericordia Veciana De Las Heras, Rayane Chami, Susana Jiménez-Murcia, José Antonio Fernández-Formoso, Janet Treasure, Fernando Fernández-Aranda

**Affiliations:** 1 Department of Psychiatry, University Hospital of Bellvitge-IDIBELL, 08907 Barcelona, Spain; maria.lozano@bellvitgehospital.cat (M.L.-M.); gtesta@idibell.cat (G.T.); cvintro@bellvitgehospital.cat (C.V.-A.); isasanchez@bellvitgehospital.cat (I.S.); nriesco@bellvitgehospital.cat (N.R.); imartinezz@outlook.es (I.M.-Z.); sjimenez@bellvitgehospital.cat (S.J.-M.); 2 CIBER Fisiopatologia Obesidad y Nutrición (CIBERobn), Instituto de Salud Carlos III, 28029 Madrid, Spain; Roser.Granero@uab.cat (R.G.); tono.fernandez@ciberisciii.es (J.A.F.-F.); 3 Addictive Behavior Unit, Department of Psychiatry, Hospital de la Santa Creu i Sant Pau, 08001 Barcelona, Spain; 4 Department of Experimental Psychology, Mind, Brain, and Behavior Research Centre, University of Granada, 18071 Granada, Spain; jcesar@ugr.es (J.C.P.); megiasrobles@gmail.com (A.M.); 5 Department of Basic Psychology, Autonomous University of Madrid, 28049 Madrid, Spain; juan.fco.navas@gmail.com; 6 Universitat Oberta de Catalunya, 08018 Barcelona, Spain; 7 Clinical Sciences Department, School of Medicine, University of Barcelona, 08907 Barcelona, Spain; 8 Department of Psychobiology and Methodology, Autonomous University of Barcelona, 08035 Barcelona, Spain; 9 Neurophysiology Unit, Neurology Department, Hospital Universitari de Bellvitge, 08907 Barcelona, Spain; mveciana@bellvitgehospital.cat; 10 Section of Eating Disorders, Institute of Psychiatry, Psychology & Neuroscience (IoPPN), King’s College London, London SE5 8AF, UK; rayane.chami@kcl.ac.uk (R.C.); janet.treasure@kcl.ac.uk (J.T.)

**Keywords:** food craving, food addiction, emotion regulation, eating disorders, anorexia nervosa, event related potentials, EEG, neurophysiology, psychopathology

## Abstract

Background: Difficulties in emotion regulation and craving regulation have been linked to eating symptomatology in patients with anorexia nervosa (AN), contributing to the maintenance of their eating disorder. Methods: To investigate clinical and electrophysiological correlates of these processes, 20 patients with AN and 20 healthy controls (HC) completed a computerized task during EEG recording, where they were instructed to down-regulate negative emotions or food craving. Participants also completed self-report measures of emotional regulation and food addiction. The P300 and Late Positive Potential (LPP) ERPs were analysed. Results: LPP amplitudes were significantly smaller during down-regulation of food craving among both groups. Independent of task condition, individuals with AN showed smaller P300 amplitudes compared to HC. Among HC, the self-reported use of re-appraisal strategies positively correlated with LPP amplitudes during emotional regulation task, while suppressive strategies negatively correlated with LPP amplitudes. The AN group, in comparison to the HC group, exhibited greater food addiction, greater use of maladaptive strategies, and emotional dysregulation. Conclusions: Despite the enhanced self-reported psychopathology among AN, both groups indicated neurophysiological evidence of food craving regulation as evidenced by blunted LPP amplitudes in the relevant task condition. Further research is required to delineate the mechanisms associated with reduced overall P300 amplitudes among individuals with AN.

## 1. Introduction

Anorexia nervosa (AN) is recognized as a severe mental disorder characterized by restrained eating, dysfunctional thoughts, preoccupation concerning food and body image disturbance [1,2]. In addition to maladaptive cognitions and behaviours, difficulties in emotion regulation and food craving regulation have been linked to disordered eating symptomatology (i.e., binging, purging, or restriction), which are considered to be contributing factors to the maintenance of eating disorders [3,4,5]. 

Emotion regulation is understood as the process by which individuals are able to modulate the way they experience and express their emotions [6]. Two strategies have been of special interest when studying emotion regulation: suppression and reappraisal. Suppression consists of inhibiting the behavioural expression of an emotional response to a stressor, while reappraisal implicates reinterpreting the meaning of an emotional event [7]. Although the former is considered to be a maladaptive response, the latter is considered to be an adaptive strategy used to reduce the impact of negative emotional states evoked during stressful situations. In this sense, reappraisal appears to be particularly effective because it implies less physiological and cognitive costs, as well as less negative impact on memory compared to suppression [8]. 

It is hardly surprising that dysfunctional emotion regulation is considered to be a key mechanism underpinning numerous psychopathologies [9,10,11,12], among which we can find the whole spectrum of eating disorders [13,14,15]. Several studies suggest that, due to emotion regulation being adopted as a means of regulating negative emotions, difficulties in this area could be involved in the development and maintenance of problematic eating disorder-related behaviours [16,17]. Accordingly, emotion dysregulation has been exhibited as a trait among patients with AN, and also as a key element of their therapy [18,19]. 

Interestingly, food craving (i.e., intense desire for specific food), which is considered a hallmark of food addiction, has been recently proposed as an affective state involving behavioural and physiological changes [20]. Food craving is not necessary followed by increasing eating [21] and can be regulated like other affective states as suggested in recent studies in the non-clinical population [22,23,24]. In the eating disorder population, food craving and the related food addiction have been frequently reported [25], with a few studies suggesting the presence of these features even in patients with AN, especially those with binging/purging symptoms [26,27]. However, to our best knowledge, there is a lack of studies investigating food craving regulation in eating disorders, including AN. 

Event-related potentials (ERPs) are electrical changes in electroencephalographic (EEG) recordings that are time-locked to sensory or cognitive events. Given the excellent time resolution, the event-related potential (ERP) technique has been adopted to investigate the time course of emotion regulation and craving regulation [28]. During late processing, the P300 component has been relevant to attention research as it increases with stimulus salience. Following it, the late positive potential (LPP) is thought to reflect motivated attention [7,29].

Previous ERP studies in the non-clinical population showed that the amplitude of the P300 and LPP components can be modulated by different emotion regulation strategies [30,31,32,33,34,35,36,37,38]. Due to the clinical relevance of emotions in daily life, numerous EEG studies have focused on down-regulation of P300 and LPP amplitudes in response to negative and positive emotions [30,31,32,33,34,35,36,37,38]. Although most studies point to a reduction of LPP amplitudes when participants try to down-regulate their negative emotions [30,31,32,33,39,40], other research studies have found no significant modulation of this component [35,38], or even a modulation in the opposite direction [41]. Focusing on the eating disorders field, several ERP studies have shown emotion regulation difficulties among individuals with comorbidities, such as anxiety disorders and alexithymia [42,43]. Nevertheless, no studies to date have examined ERP modulations by emotion regulation in specific eating disorder populations such as AN. 

On the other hand, several ERP studies have strived to demonstrate the efficacy of different emotion regulation techniques in modulating food craving in healthy individuals. For instance, using reappraisal in order to change the emotional meaning of food increased LPP amplitude when participants tried to focus on the long-term consequences of eating high-caloric food [44]. Reappraisal was also employed in another study in which participants were instructed to increase or decrease the appetitive value of food. Results showed that P300 and LPP amplitudes to food cues were larger when participants tried to increase the appetitive value of food in comparison to the condition of decreasing or just watching the images [45]. Moreover, research instructing restrained eaters to either reappraise cravings, suppress cravings, or watch food during a food task found that engaging in cognitive reappraisal or suppression significantly reduced ERP amplitudes compared to the food watch condition [46]. Although research has demonstrated the efficacy of emotion regulation techniques in normal-weight healthy individuals, up to date there is a lack of ERP research assessing regulation of food craving in AN patients [47]. Elucidating neurophysiological mechanisms of food craving regulation could pave the way for new treatment approaches for anorexia nervosa, in which emotion regulation techniques might be employed to alter the motivational value of certain foods.

The primary aims of the study were to explore clinical and electrophysiological features of emotion regulation and food craving regulation among patients with AN. As for the clinical profile, we hypothesized that individuals with AN would present higher self-reported emotion dysregulation and food addiction compared to a group of healthy control (HC). Regarding electrophysiological data, we hypothesize that there will be a significant reduction in LPP amplitudes during conditions requiring participants to down-regulate negative emotions or food craving, as opposed to neutral conditions. Based on previous clinical research reporting emotion and food craving regulation difficulties in AN, we also aim to explore between-group differences in ERP during down-regulation of emotion or food craving. Finally, we explored to which extent self-reported emotion regulation strategies (adaptive or maladaptive) correlates with ERP (i.e., P300, LPP) during down-regulation of food craving or negative emotions. Maladaptive strategies are expected to be predominant in AN and possibly correlate with brain response during down-regulation of emotions/food craving. 

## 2. Materials and Methods

### 2.1. Participants

The present study involved two different groups: a clinical group of patients with anorexia nervosa (AN) and a healthy control group (HC). The AN clinical group was comprised of 20 female treatment-seeking patients diagnosed with AN (60% AN restrictive subtype, 40% AN binge/purging subtype) according to DSM-5 criteria (Body Mass Index (BMI) < 18.5) [48]. Recruitment was conducted at the Eating Disorders Unit within the Department of Psychiatry at Bellvitge University Hospital, a public health hospital certified as a tertiary care centre with a highly specialised unit for the treatment of eating disorders in Barcelona (Spain). The HC group consisted of 21 female participants who had no history of an eating disorder. Participant groups were matched by age and education level. All participants were recruited between June 2016 and July 2018.

Data from one healthy control participant had to be excluded due to poor EEG data quality. The final sample size consisted of 40 participants, of whom 20 were patients with AN (mean age = 22.7 years, SD = 6.51, age range 18 to 43, mean BMI = 16.6 kg/m^2^, SD = 1.1), and 20 were HC (mean age = 21.0 years, SD = 5.12, age range 18 to 39; mean BMI = 20.7 kg/m^2^, SD = 1.78). Among AN group, 9 patients (45%) reported psychotropic treatment (antidepressants: n = 4, 20%; anxiolytics: n = 1 5%; both: n = 4, 20%). Exclusion criterion for all participants were: (a) being male, (b) younger than 18 years, (c) current or life-time history of chronic illness or neurological condition (abnormal EEG activity), which could influence electrophysiology and/or the neuropsychological assessment, (c) lifetime diagnosis of a severe mental health condition (bipolar disorder, lifetime diagnosis of psychotic disorder), (d) current substance dependence or any other mental disorder that could interfere cortical activity or the assessment. Additionally, in the HC group, an exclusion criteria was a lifetime diagnosis of any eating disorder, assessed by means of the Mini International Neuropsychiatric Interview (MINI) [49], being overweight/obese (Body Mass Index (BMI) ≥ 25), or underweight (BMI < 18.5).

Written informed consent was obtained before participation in the study, which was approved by the Ethics Committee of University Hospital of Bellvitge in accordance with the Helsinki Declaration of 1975 as revised in 1983. Participants received no compensation for taking part in the study.

### 2.2. Procedure

Patients who sought treatment for AN as their primary health concern were assessed by an experienced clinical psychologist as part of the Eating Disorders Unit protocol, which is based on DSM-5 criteria and includes height and weight measurements. All patients consecutively diagnosed with AN were screened for the inclusion criteria of the study and gave informed consent for voluntarily accepting to be part of the study. HC participants were recruited within a university campus and, if they were interested in taking part in the study, an eligibility screening was conducted prior to the initial face-to-face assessment session. 

The variables explored in the present study were assessed in two separate sessions of approximately 90 minutes each. Firstly, participants were evaluated with the MINI to exclude those patients with any severe psychiatric condition. Afterwards, they completed a battery of self-reported questionnaires (DERS, ERQ, SCL-90-R, YFAS-2). Next, participants performed the experimental tasks (food craving and emotion regulation) during EEG acquisition. Participants were instructed to have a ‘normal’ meal 90 minutes before the session and then to refrain from eating or drinking coffee. Additional information was collected on the day of the experimental session, in order to control for a set of variables (i.e., food consumed on the day of the session, menstrual cycle, and alcohol or drugs consumption in the last 24h). In a second session, participants completed a different set of experimental neurophysiological tests (data will be reported in separate manuscript). 

### 2.3. Clinical Assessment

The *Mini-International Neuropsychiatric Interview* (MINI) [49] is a short structured diagnostic interview for the major psychiatric disorders in DSM-III-R [50], DSM-IV [51] and DSM-5 [16] and ICD-10 [52]. Validation and reliability studies have been done comparing the MINI to the Structured Clinical Interview (SCID-P) [53] based on DSM-III-R [50] and the Composite International Diagnostic Interview (CIDI) [54], which is a structured interview developed by the World Health Organization. These studies showed that the MINI has similar reliability and validity properties to both instruments. With an administration time of approximately 15 minutes, it was designed to meet the needs for a short, yet accurate, structured psychiatric interview for multicentre clinical trials and epidemiology studies and to be used as a first step in outcome tracking in non-research clinical settings. The standard MINI assesses the 17 most common disorders in mental health. The disorders were selected based on current prevalence rates of 0.5% or higher in the general population in epidemiology studies. In the interest of brevity, it uses branching tree logic. 

Difficulties in Emotion Regulation Scale (DERS; Spanish validation) [15,55,56] is a 36-item self-report scale that assesses relevant difficulties in emotion regulation on six subscales: non-acceptance of emotional responses, difficulties engaging in goal directed behaviour, impulse control difficulties, lack of emotional awareness, limited access to emotion regulation strategies and lack of emotional clarity. The measure yields a total score as well as scores on the six subscales. Higher scores indicate greater problems with emotion regulation. Cronbach’s α for the total score in the present study was 0.91.

*Emotion Regulation Questionnaire, Spanish version* (ERQ) [57] is a 10-item questionnaire to assess the respondents’ tendency to implement two emotion regulation strategies: reappraisal and emotional suppression. For the present study it shows a Cronbach’s α of 0.76 for the suppression scale, and 0.85 for the reappraisal scale.

*Symptom Checklist-90 Revised* (SCL-90; Spanish validation) [58,59] is a 90-item questionnaire which evaluates psychopathological symptoms. It also includes a global severity index (GSI), designed to measure overall psychological distress. Internal consistency for GSI scale in the present study sample was 0.98.

*The Yale Food Addiction Scale Version 2.0* (YFAS-2) [25] is a 25 item self-report questionnaire to measure addictive food behaviours. It consists of seven scales which refer to the criteria for substance dependence: (1) tolerance, (2) withdrawal, (3) substance taken in larger amount/period of time than intended, (4) persistent desire/unsuccessful efforts to cut down, (5) great deal of time spent to obtain substance, (6) important activities given up to obtain substance, (7) use continued despite psychological/physical problems. The Cronbach’s α value for the present study was 0.97.

### 2.4. Electrophysiological Assessment

Participants completed an emotion regulation task and a food craving regulation task during continuous EEG recording. 

*Emotion regulation task:* The task stimuli consisted of 180 images, of which 120 were negative images distributed in two blocks of 60 images each and 60 were neutral images grouped in a third block. Stimuli were presented for 3000 ms, with an inter-trial interval ranging from 3500 ms to 4500 ms. Negative images and neutral images were matched on contrast, brightness, resolution and complexity. Images were taken from the International Affective Picture System (IAPS) [60] and each image was presented only once during the task. Stimulus presentation was carried out by Presentation® software (Version 16.0) [61]. Participants were seated approximately 60 cm in front of a computer screen and the images were shown serially and occupied 35.1° of visual angle horizontally and 28.1° vertically. 

For negative images, participants were instructed to either view each picture and allow themselves to feel any emotional response it might elicit (from now on referred to as Observe Negative) or to view each picture and try to reduce the emotional response that it might elicit (from now on referred to as Regulate Negative). For neutral images, participants were instructed to view each picture and allow themselves to feel any emotional response it might elicit (from now on referred to as Observe Neutral) while viewing the images and feeling the elicited emotion. 

*Food craving regulation task:* Task stimuli consisted of 180 images, of which 120 were highly palatable food images distributed in two blocks of 60 images each and 60 were neutral images (i.e., office items) grouped in a third block. Stimuli were presented for 3000 ms, with an inter-trial interval ranging from 3500 ms to 4500 ms. Food images and neutral images were matched on contrast, brightness, resolution and complexity. Images were taken from Food Pics [62] and each image was presented only once during the task. Stimulus presentation was carried out by Presentation® software (Version 16.0) [61]. Participants were seated approximately 60 cm in front of a computer screen. The images were shown serially and occupied 18.9° of visual angle horizontally and 17.1° vertically. 

For food images, participants were instructed to either view each picture and allow themselves to feel any emotional response it might elicit (from now on referred to as Observe Negative) or to view each picture and try to reduce the emotional response that it might elicit (from now on referred to as Regulate Negative). For neutral images, participants were instructed to view each picture and allow themselves to feel any emotional response it might elicit (from now on referred to as Observe Neutral).

### 2.5. Electrophysiological Recording and Analysis

The electroencephalogram (EEG) was recorded continuously throughout the experimental task using PyCorder (BrainVision). 60 active Ag/AgCI electrodes were inserted into an EEG recording cap (EASYCAP GmbH), distributed after the 10–20 system; additional three electrodes were adopted for recording vertical and horizontal electrooculogram (EOG) and Cz was used as online reference. Impedances were kept below 20 KOhm using the SuperVisc high-viscosity electrolyte gel for active electrodes. Signals from all channels were digitized with a sampling rate of 500 Hz and 24 bit/channel resolution and online filtered between 0.1 and 100 Hz. 

Offline EEG analyses were performed with Brain Vision Analyzer (Version 2.2.0) [63] consisting of the following steps: high pass filtering 0.1 Hz, low pass filtering at 30 Hz (Butterworth zero phase filter; 24 dB/octave slope) and notch filter at 50 Hz; raw data inspection for manual detection of artefact and screening for bad channels, semi-automatic eye-blink correction using independent component analysis (ICA); artefact rejection of trials with an amplitude exciding ± 80 µV; and baseline correction adopting the pre-stimulus interval between −200 and 2000 ms. EEG data were segmented into 2200 ms epochs from 200 ms before to 2000 ms after stimulus onset. Data were baseline corrected against the mean voltage during the −200 pre stimulus period. Artefact free epochs were separately averaged for each subject in each experimental condition for each paradigm. 

ERP analyses were based on visual inspection of the grand average waveforms and the existing literature [45,46]. ERP components were analysed in a central-parietal cluster (CP1, CP5, P3, P7, CP2, CP6, P4, P8). P300 mean amplitude (μV) was computed in the time-window between 280 and 400 ms; LPP mean amplitude (μV) was measured within two time-windows: at 500-1000 ms (LPP1) and 1000-1500 ms (LPP2) [64,65,66].

### 2.6. Statistical Analysis

Statistical analysis was carried out with Stata Statistical Software: Release 15 for Windows [67]. The variables of the study (ERQ, YFAS, DERS and SCL-90-R) were compared between groups using t-tests for quantitative measures and chi square (χ^2^) tests for categorical measures. Comparisons were considered significant with *p* < 0.05 after Bonferroni-Finner correction to avoid Type-I errors (Finner, 1993). The effect size for the mean differences/proportions was measured through Cohen’s-*d* coefficient (low/small effect size was considered for |*d*| > 0.2, moderate for |*d*| > 0.5 and large/high for |*d*| > 0.8; Kelly and Preacher, 2012). In this study, different dimensional and categorical measures for the YFAS 2.0 were analysed: firstly, the YFAS 2.0 dimensional symptom count, which measures the 11 DSM-5 SRAD criteria (raw scores are in the range of 0–11); and secondly, the categorical classification based on the dimensional symptom count, a threshold for food addiction (presents for individuals with at least two symptoms plus self-reported clinically significant impairment or distress, and absent for participants who did not meet these criteria). The capacity of the dimensional YFAS 2.0 symptom count to discriminate between the groups was tested through two sample T-test, and the capacity of the YFAS 2.0 categorical classifications to discriminate between the diagnostic sub-types was tested through chi-square tests (χ^2^).

The mean amplitudes (μV) of the emotion regulation and food craving regulation tasks were analysed for each ERP component (P300, LPP1, LPP2) with independent 3 × 2 mixed design analyses of variance (ANOVA), with condition as the within-subject variable (Regulate Negative/Food, Observe Negative/Food, Observe Neutral) and group as the between subject variable (HC versus AN). Pairwise comparisons were used to follow up main effects (for non-significant interaction condition-by-group) and single effects (for significant interaction condition-by-group). 

Pearson’s correlations were calculated for each group to estimate correlations between ERPs in the “regulation” condition of the emotion/food craving regulation tasks and ERQ subscales (ERQ-suppression; ERQ-reappraisal). Due to the strong association between this model and the sample size, practical relevance was based on the own coefficient measure (effect size was considered low/poor for |R| > 0.10, moderate for |R| > 0.24 and large/high for |R| > 0.37) [66].

## 3. Results

### 3.1. Comparison of Clinical Profiles

There were no significant between-group differences in age (*p* = 0.364, |*d*| = 0.29). As expected, the HC group had significantly greater BMIs (*p* < 0.001, |*d*| = 2.79), lower mean scores on psychopathological self-report measures (i.e., the SCL-90-R GSI, DERS and YFAS), and higher mean scores on ERQ-Reappraisal. The prevalence of participants with food addiction positive screening score was also higher in the AN group (70% vs. 0%, *p* < 0.001, |*d*| = 2.16) (See Table 1). When comparing food addiction between AN sub-types, significant higher scores were displayed by the AN-BP subtype on the YFAS total score (*p* = 0.031, |*d*| = 1.00) and in all the YFAS criteria with exception of “withdrawal symptoms” (See Table 2).

### 3.2. ERP Results: Emotion Regulation Task

P300. The mixed design ANOVA yielded a significant main effect of condition (Regulate Negative, Observe Negative, Observe Neutral; F:27.7, df = 2/38, *p* < 0.001; η^2^ = 0.421) and a significant main effect of group (HC versus AN; F = 10.9, df = 1/38, *p* = 0.002; η^2^ = 0.223). No significant group x condition interaction was detected (F = 1.51, df = 2/37, *p* = 0.229; η^2^ = 0.038). Post-hoc t-tests revealed that the main effect of condition was due to higher P300 mean amplitude in Observe Negative and in Regulate Negative conditions compared to the neutral one (Observe Negative vs. Observe Neutral *p* < 0.001; Regulate vs. Observe Neutral *p* < 0.001). With regards to the main effect of group, the AN group showed significantly smaller mean P300 amplitudes compared to HC group (*p* = 0.002).

LPP1. The mixed design ANOVA showed a significant main effect of condition (F = 51.7, df = 2/38, *p* < 0.001; η^2^ = 0.577), but no significant main effect for group (F = 3.04, df = 1/38 *p* = 0.089; η^2^ = 0.074) or group x condition interaction (F = 1.01, df = 2/37, *p* = 0.369; η^2^ = 0.026). Post hoc t-tests for the main effect of condition showed higher LPP1 amplitudes in the Observe Negative and Regulate Negative conditions, compared to Neutral condition (Observe Negative vs. Observe Neutral *p* < 0.001; Regulate vs. Observe Neutral *p* < 0.001).

LPP2. The mixed design ANOVA showed a significant main effect of condition (F = 13.1, df = 2/38, *p* < 0.001; η^2^ = 0.256), but no main significant effect of group (F = 0.22, df = 1/38, *p* = 0.643; η^2^ = 0.006) or group x condition interaction (F = 0.05, df = 2/37, *p* = 0.954; η^2^ = 0.001). Post-hoc t-tests revealed that the effect of condition was due to higher mean LPP2 amplitudes in both the Observe Negative and Regulate Negative conditions, compared to the neutral one (Observe Negative vs. Observe Neutral *p* = 0.002; Regulate Negative vs. Observe Neutral *p* < 0.001). 

**Figure 1:**
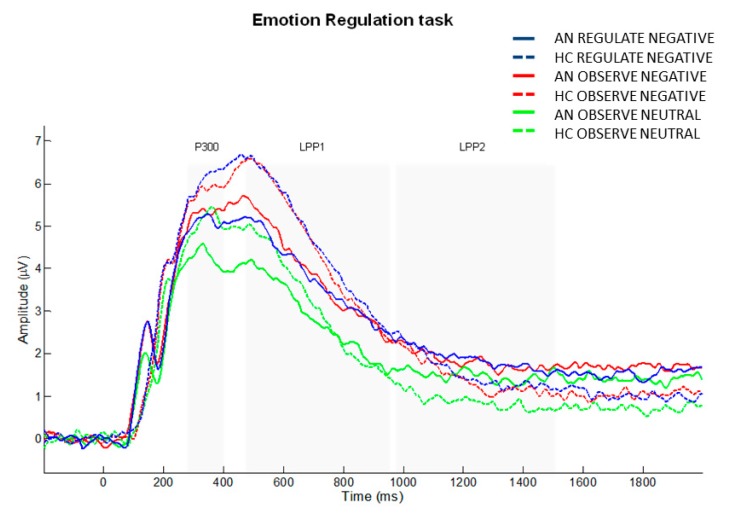
Grand average waveforms of the ER task, for each experimental condition (Regulate Negative, Observe Negative, Observe Neutral) and group (HC, AN), in the centro-parietal cluster of electrodes.

Means and standard deviations of the ERP amplitudes (μV) for each component (P300, LPP1, LPP2) are reported in Table 3 (see also Figure 1). 

### 3.3. ERP Results: Food Craving Regulation Task

P300. The mixed design ANOVA showed a significant main effect of condition (Regulate Food, Observe Food, Observe Neutral; F = 47.2, df = 2/38, *p* < 0.001; η^2^ = 0.560) and a significant main effect of group (HC versus AN; F = 6.72, df = 1/38, *p* = 0.014; η^2^ = 0.154), but no significant group x condition interaction (F = 1.40, df = 2/37, *p* = 0.252; η^2^ = 0.037). Post-hoc t-tests for the main effect of condition showed higher amplitude in Observe Food and Regulate Food compared to the Observe Neutral condition (Observe Food vs. Observe Neutral *p* < 0.001; Regulate Food vs. Observe Neutral *p* < 0.001). Moreover, the AN group showed significantly smaller mean P300 amplitudes compared to the HC group (*p* = 0.014). 

LPP1. The mixed design ANOVA showed a significant main effect of condition (F = 38.5, df = 2/38, *p* < 0.001; η^2^ = 0.504), but no significant main effect of group (F = 0.73, df = 1/38, *p* = 0.397, η^2^ = 0.019) or a significant group x condition interaction (F = 0.25, df = 2/37, *p* = 0.778; η^2^ = 0.007). Post-hoc t-tests for condition revealed higher LPP1 in both Observe Food and Regulate Food compared to the Observe Neutral condition (Observe Food vs. Observe Neutral *p* < 0.001; Regulate Food vs. Observe Neutral *p* < 0.001), and higher LPP1 in Observe Food compared to Regulate Food (*p* = 0.040). 

LPP2. The mixed design ANOVA showed a significant main effect of condition (F = 23.3, df = 2/38, *p* < 0.001; η^2^ = 0.380), but no significant main effect of group (F = 0.13, df = 1/38, *p* = 0.911, η^2^ = 0.001) or group x condition interaction (F = 0.10, df = 2/37, *p* = 0.906, η^2^ = 0.003). Post-hoc t-tests for condition revealed higher LPP1 in both Observe and Regulate compared to the Observe Neutral condition (Observe Food vs. Observe Neutral *p* < 0.001; Regulate Food vs. Regulate Neutral *p* < 0.001), and higher LPP1 in Observe Food compared to Regulate Food (*p* = 0.008). 

**Figure 2:**
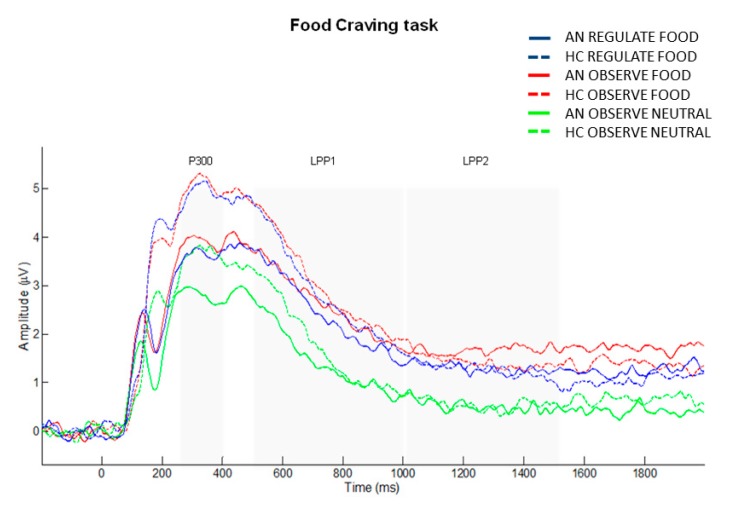
Grand average waveforms of the FRC task, for each experimental condition (Regulate Food, Observe Food, Observe Neutral) and group (HC, AN), in the centro-parietal cluster of electrodes.

Mean and standard deviations of the ERP amplitudes (μV) for each component (P300, LPP1, LPP2) are reported in Table 4 (see also Figure 2). 

### 3.4. Correlations between ERPs and Self-reported Emotional Regulation Strategies

Emotion Regulation Task and ERQ. In the HC group, reappraisal, as measured using the ERQ, was positively correlated with mean LPP1 amplitudes, while suppression was negatively correlated with mean LPP2 amplitudes. No significant correlations were found in the AN group.

Food Craving Regulation Task and ERQ. ERQ-reappraisal was positively correlated with mean LPP2 in the HC group, but not in the AN group. ERQ-suppression was negatively correlated with mean LPP1 and LPP2 amplitudes among patients with AN, but not in the HC group. 

Table 5 shows the correlation matrix measuring the correlation between self-report measures of emotion regulation strategies (ERQ-suppression; ERQ-reappraisal) and ERPs amplitudes during emotion regulation (Regulate Negative) and food craving regulation (Regulate Food). 

## 4. Discussion

In the present study, clinical and electrophysiological features of emotion regulation and food craving regulation among patients with AN were investigated by means of self-report and ERP measures.

Results from self-report measures of emotion regulation, confirmed greater difficulties in emotion regulation in patients with AN compared to the HC group (as suggested by DERS scores). This is in line with previous studies comparing AN with HC using the same questionnaire [5,68,69,70]. In addition, in the ERQ subscales, differences between groups were found, suggesting that patients with AN most frequently implemented maladaptive strategies (i.e., suppression) than adaptive strategies (i.e., reappraisal). This latter results corroborated previous findings suggesting dysfunctional emotion regulation strategies (e.g. suppression, avoidance) in populations with eating disorder [71,72,73], as is the case with other psychiatric disorders [74]. Moreover, problematic eating behaviours, such as binging, purging, and restriction, can be seen as maladaptive strategies to avoid or suppress negative emotions [68,75,76]. With regards to food addiction, a higher score was detected in the AN, as opposed to the HC group. Additional comparisons within the AN sub-types suggested higher scores in multiple dimensions of food addiction in AN-BP compared to AN-R. The present findings portray evidence of the relevance of food addiction to AN, specifically in patients with binging/purging symptoms. It is important to note that food addiction scores have been more typically described in patients with binge-subtype eating disorder [77,78,79,80], with some inconclusive or less evident results in AN. In a previous study exploring food addiction in eating disorders, patients with AN binge/purging subtype showed the highest prevalence of food addiction although half of the AN patients with restrictive type also positively scored for food addiction [27]. 

Results from electrophysiological measures collected in the emotional regulation task indicated enhanced mean P300 and LPP amplitudes in presence of pictures depicting negative emotions compared to neutral pictures in both AN and HC groups. This suggested enhanced processing of emotional stimuli, potentially due to their evolutionary salience, in accordance with previous ERP literature on ‘healthy’ populations [7,81,82,83,84,85]. Based on our results, we can suggest that, similarly to HC, patients with AN display a facilitated processing of stimuli with negative emotional valence. Although a previous ERP study reported altered processing of emotional stimuli in patients with AN [86], these controversial findings could be explained by the use of different types of stimuli and task (i.e., recognition of emotional faces).

Despite of the reported ERP indices of emotional processing, the instruction to down-regulate negative emotions did not elicit significant differences in mean P300 and LPP amplitudes when compared to passive viewing of negatively valenced emotional stimuli in any group. Since a reduction in LPP amplitude has been previously shown during emotion down-regulation in healthy population [30,31,32,33,39,40], the lack of this effect can be explained by a failure in emotion down-regulation that occurred in both AN patients and controls. This can be due to the fact that participants were not instructed to adopt a specific regulation strategy (e.g. reappraisal; suppression), which makes it more difficult to successfully achieve emotion regulation. However, adopting visual analogue scales to measure self-reported down-regulation is necessary to avoid premature conclusions. 

During the food craving regulation task, pictures of food elicited greater mean P300 and LPP amplitudes compared to neutral non-food pictures in both AN and HC groups. This can be interpreted as motivated attention, meaning a higher amount of attentional resources allocated to process food stimuli [87]. However, we did not find higher motivated attention toward food in patients with AN when compared to HC, suggesting similar allocation of attentional resources toward food-stimuli, at latest stages of attentional processing. This is in accordance with a previous study in which patients with AN did not display enhanced P300/LPP toward high-caloric food, but only for low-caloric food pictures when compared with HC [88]. Since we were interested in investigating regulation of food craving, which is generally experienced in response to “forbidden foods” (i.e., high caloric), low-caloric food was not included in our study. 

Interestingly, smaller LPP amplitudes were detected during down-regulation of craving compared to passing viewing food pictures, possibly suggesting successful down-regulation of food craving in both groups. This result is in line with a previous study in non-clinical ‘restrained’ eaters, showing that P300 and LPP amplitudes were reduced during down-regulation of food craving compared to the passive viewing of food-related pictures [46]. As the first ERP study which explores food craving regulation in patients with AN, we could observe that, despite AN reported greater “food addiction” symptomatology, these subjects were able to regulate food craving, as depicted at a neurophysiological level. Nevertheless, differential ERP response during food craving regulation may be expected between AN-BP and AN-R. Thus, further research in larger sample sized including different AN sub-types is needed to deeply understand the neurophysiological mechanisms underpinning this craving modulation in AN.

Finally, differences in ERP between patients and controls were depicted by smaller P300 amplitudes in the AN group. This overall reduction in mean P300 amplitudes was consistent in both tasks and regardless of experimental condition. Reduced neurophysiological response in AN could reflect neurocognitive alterations, possibly as a secondary effect of malnutrition which consequently affect cognitive functioning [89]. Accordingly, cognitive difficulties have been suggested in patients with AN, especially in memory, attention and executive functions (i.e. decision-making, set-shifting [90,91,92]. Similarly to our findings, previous ERP studies adopting different tasks showed reduced P300 in AN compared to controls, regardless of the emotional relevance of the stimuli [93,94].

Exploratory correlations in each group were performed in order to explore how emotion regulation strategies modulate both emotion and food craving regulation at a neurophysiological level. As for the emotion regulation task, our findings suggest that, only among HC, the tendency to suppress emotions correlated with larger LPP amplitudes, while the tendency to reappraise emotions correlated with lower LPP amplitudes. This may suggest that the tendency to adopt different emotion regulation strategies (i.e. reappraisal or suppression) is related with different modulation of the LPP amplitude while regulating emotions, at least in healthy individuals. Since the modulation of LPP amplitude has been linked to reappraisal of negative emotions in HC [30,31,32,33,39,40], the present results may further suggest a link between neurophysiological markers of emotion regulation and the tendency to adopt reappraisal as cognitive strategy to down-regulate negative emotions in the non-clinical population. By contrast, LPP response did not significantly correlate with emotion regulation strategies among patients with AN.

Similarly to the emotion regulation task, the LPP amplitude during down-regulation of food craving was positively related to ERQ-reappraisal in HC. By contrast, LPP amplitudes negatively correlated with ERQ-suppression in patients with AN. These latter results could suggest that neurophysiological response during down-regulation of food craving is related to different emotion regulation strategies in patients as compared to controls, which is in line with the differences observed in ERQ scores among groups. Interestingly, significant correlations with suppression in AN were specifically present in the food craving regulation task, and this can be linked to the fact that patients tend to adopt dysfunctional eating behaviours (e.g. bingeing/purging, restriction) as maladaptive strategies to regulate negative emotions, as showed by higher scores in ERQ-suppression. 

It is important to consider some limitations when interpreting the results of the present study. Firstly, our sample size is rather low, which might have decreased the likelihood of detecting a significant difference if it existed [95]. Further studies with larger samples would be required to confirm our findings. Moreover, the small size of the sample did not allow us to distinguish and compare restrictive and purging AN sub-types. Given that different AN sub-types may exhibit different neurobiological correlates [96], future studies with larger samples should explore neural correlates of emotion regulation and food craving in different AN sub-types. In addition, our sample only consisted of female participants, which limits the generalizability of the results to a wider population. Additionally, we did not expose individuals to real food stimuli, which would have mimicked real-life situations and perhaps elicited stronger emotional and physiological reactions than food pictures [97]. Given the nature of the paradigms, another limitation of the study is the lack of eye-tracking and the lack of arousal tracing. Additional studies should further control eye-movements and attention focus during the image presentation. Furthermore, a proportion of patients with AN were under psychopharmacological medication (i.e., antidepressants, neuroleptic drugs, and benzodiazepines) and our sample did not allow us to control for medication. Finally, the present study design is cross-sectional in nature and claims regarding causality cannot be made. Future longitudinal studies are required to examine the extent to which the repetitive use of emotion regulation and food craving regulation techniques might modify the long-term neurophysiological responses in AN patients.

## 5. Conclusions

To conclude, previous ERP findings did not appear to mirror clarifying findings regarding eating disorders’ aetiology and functioning. Therefore, to this date, they might not be used as accurate parameters or biomarkers that could be directly employed in the diagnosis or treatment of eating disorders [98]. To our knowledge, this is the first study which has examined the electrophysiological features of emotion and food craving regulation among patients with AN. Interestingly, ERP results suggest a successful down-regulation of food craving in AN, despite the fact that AN reported greater food addiction symptomatology. Nevertheless, further research including different AN sub-types is needed to deeply understand the neurophysiological mechanisms underpinning this craving modulation in AN.

Furthermore, although ERP did not depict differential response between AN and HC while down-regulating emotions or food craving, reduced P300 mean amplitudes were detected in AN when compared to HC. This result might reflect a general alteration in the neurophysiological responses of AN patients, which is possibly related to their prolonged state of malnutrition [99]. In this regard, this study provides an objective parameter of those impairments which long-lasting malnutrition might be occasioning in the neural systems of AN patients. Previous research has also found neurophysiological dysfunctions in AN, which do not always seem to be normalised after weight gain [98]. In that respect, it would be of great interest that future studies explore not only if neurophysiological alterations remain or, on the contrary, are ameliorated after patients’ recovery, but also investigate the factors which might contribute to normalise neural responses in AN (e.g., weight gain, pharmacological treatments, specific psychological interventions, etc.).

On the other hand, clinical measures showed that patients with AN were characterized by food addiction symptoms and difficulties in emotion regulation with the tendency to use maladaptive techniques (i.e., suppression) to manage negative emotions. Moreover, this is the first study which relates the use of suppression strategies to smaller ERP amplitudes during food craving regulation in AN patients. This possibly reflects their tendency to adopt dysfunctional eating behaviours as maladaptive strategies to regulate negative emotions. Future interventions should focus on implementing more effective emotion regulation techniques such as reappraisal, which act through a reinterpretation of emotional situation in order to reduce its emotional impact. Reappraisal has shown a better capacity to decrease negative emotional experience, consequently reducing distress [100]. 

Further research with larger samples and considering AN sub-types is needed to deeply understand the neurophysiological mechanisms underpinning emotion and food craving modulation in AN. 

## Figures and Tables

**Table 1:** Comparison of the clinical profile between groups.

	HC (*n* = 20)		AN (*n* = 20)		*T-stat*	*p*	*|d|*
	*Mean*	*(SD)*	*Mean*	*(SD)*			
Age (years-old)	21.00	(5.12)	22.70	(6.51)	0.92	0.364	0.29
BMI (current, kg/m^2^)	20.72	(1.78)	16.63	(1.06)	8.82	<0.001 *	**2.79 ^†^**
SCL-90-R: GSI score	0.65	(0.45)	1.59	(0.70)	5.10	<0.001 *	**1.61 ^†^**
DERS: Total score	73.30	(16.12)	114.25	(23.36)	6.45	<0.001 *	**2.04 ^†^**
ERQ: Reappraisal	33.50	(5.94)	24.25	(6.69)	4.62	<0.001 *	**1.46 ^†^**
ERQ: Suppression	13.45	(5.71)	15.75	(4.64)	1.40	0.170	**0.51 ^†^**
YFAS2 total score	0.75	(1.12)	4.35	(3.73)	4.13	<0.001 *	**1.31 ^†^**
	*n*	(*%*)	*n*	(*%*)	χ^2^	*p*	*|d|*
FA positive screening (YFAS-2)	0	(0.0%)	14	(70.0%)	21.54	<0.001 *	**2.16 ^†^**

Note. SD: standard deviation. HC: healthy control. AN: anorexia. FA: food addiction. * Bold: significant parameter (.05 level). ^†^ Bold: effect size into the mild/moderate (|*d*| > 0.80) to large/good range (|*d*| > 0.80).

**Table 2:** Comparison of the FA measures between AN sub-types.

	AN-R (*n* = 12)		AN-BP (*n* = 8)		χ^2^	*p*	*|d|*
	*n*	(*%*)	*n*	(*%*)			
Substance taken in larger amount	4	33.3%	4	50.0%	0.56	0.456	0.34
Persistent desire	3	25.0%	4	50.0%	1.32	0.251	**0.53 ^†^**
Much time-activity to obtain, use, recover	5	41.7%	6	75.0%	2.15	0.142	**0.72 ^†^**
Social or occupational affectation	7	58.3%	7	87.5%	1.94	0.163	**0.69 ^†^**
Use continues despite consequences	4	33.3%	5	62.5%	1.65	0.199	**0.61 ^†^**
Tolerance	0	0.0%	5	62.5%	10.00	0.002 *	**1.83 ^†^**
Withdrawal symptoms	5	41.7%	5	62.5%	0.83	0.361	0.43
Continued use despite social problems	1	8.3%	4	50.0%	4.44	0.035 *	**1.03 ^†^**
Failure to fulfil major rule obligations	1	8.3%	4	50.0%	4.44	0.035 *	**1.03 ^†^**
Use in physically hazardous situations	3	25.0%	4	50.0%	1.32	0.251	**0.53 ^†^**
Craving, or a strong desire or urge to use	2	16.7%	4	50.0%	2.54	0.111	**0.76 ^†^**
Clinically significant impairment-distress	8	66.7%	7	87.5%	1.11	0.292	**0.51 ^†^**
FA positive screening score	8	66.7%	6	75.0%	0.16	0.690	0.18
	*Mean*	*(SD)*	*Mean*	*(SD)*	*T-stat*	*P*	*|d|*
FA dimensional (YFAS2 total)	2.92	2.39	6.50	4.47	2.34	0.031 *	**1.00 ^†^**

Note. AN-R: anorexia restrictive subtype. AN-BP: anorexia bulimic-purgative subtype. FA: food addiction. SD: standard deviation. * Bold: significant parameter (.05 level). ^†^ Bold: effect size into the mild/moderate (|*d*| > 0.80) to large/good range (|*d*| > 0.80).

**Table 3:** Mean (SD) amplitudes (μV) of P300, LPP1 and LPP2 during the emotion regulation task.

	HC (*n* = 20)		AN (*n* = 20)	
	Mean	(SD)	Mean	(SD)
P300:				
observe negative	6.53	(2.72)	4.85	(1.97)
regulate negative	6.96	(2.93)	4.44	(1.84)
observe neutral	5.17	(1.86)	3.10	(1.44)
LPP1:				
observe negative	4.71	(2.59)	3.69	(1.63)
regulate negative	4.81	(2.27)	3.65	(1.49)
observe neutral	2.34	(1.53)	1.83	(1.20)
LPP2:				
observe negative	1.77	(2.16)	2.03	(1.48)
regulate negative	2.04	(2.04)	2.16	(1.11)
observe neutral	0.86	(1.47)	1.08	(1.10)

Note. HC: healthy control. AN: anorexia. SD: standard deviation.

**Table 4:** Mean (SD) amplitudes (μV) of P300, LPP1 and LPP2 during the food craving regulation task.

	HC (*n* = 20)		AN (*n* = 20)	
	Mean	(SD)	Mean	(SD)
P300:				
observe food	5.23	(2.39)	3.82	(1.47)
regulate food	5.60	(2.64)	3.67	(1.52)
observe neutral	3.68	(2.46)	2.32	(1.13)
LPP1:				
observe food	3.20	(1.91)	2.73	(1.38)
regulate food	2.86	(2.14)	2.38	(1.38)
observe neutral	1.49	(1.59)	1.25	(0.97)
LPP2:				
observe food	1.71	(1.45)	1.75	(1.19)
regulate food	1.26	(1.62)	1.21	(1.07)
observe neutral	0.42	(1.24)	0.54	(0.83)

Note. HC: healthy control. AN: anorexia. SD: standard deviation.

**Table 5:** Pearson’s correlation between the amplitudes (μV) of the P300, LPP1, LPP2 during the “regulate” condition of the emotion regulation and the food craving regulation tasks.

	Emotion Regulation Task				Food Craving Regulation Task			
	HC (*n* = 20)		AN (*n* = 20)		HC (*n* = 20)		AN (*n* = 20)	
	ERQ reappr.	ERQ suppr.	ERQ reappr.	ERQ suppr.	ERQ reappr.	ERQ suppr.	ERQ reappr.	ERQ suppr.
P300	0.003	−0.195	0.119	−0.051	0.201	−0.187	0.101	0.018
LPP1	**0.247 ^†^**	−0.205	0.130	−0.103	0.144	−0.207	0.204	**−0.258 ^†^**
LPP2	0.196	**−0.281 ^†^**	0.173	−0.058	**0.396^†^**	−0.129	0.215	**−0.370 ^†^**

Note. HC: healthy control. AN: anorexia. ^†^ Bold: effect size into the mild/moderate (|R| > 0.24) to large/good range (|R| > 0.37). Sample size: Healthy control = 20; Anorexia = 20.
